# The Conquest of Venereal Disease

**Published:** 1920-07-31

**Authors:** 


### "The Conquest of Venereal Disease."

During the last few weeks, following the Health Minister's reception of the suggestion to make the sale of preventive materials an official procedure, there has been, in our own and other correspondence columns, a decided tendency to renew the apparently interminable controversy between those who support real practical preventive education and those who draw back in fear of its moral effect. At this time, however, there comes to our notice a valuable little pamphlet written by Dr. Charles Russ,* a recognised authority on the treatment of venereal disease and its problems. He sets out to give, and indeed does give, a few plain facts which may assist the cause of progress in a conquest which most of us desire, those who enter so readily and hotly into the venereal controversy might be well advised to consult and understand them. As a review of the practical means of conquest which are available in Great Britain, we can heartily recommend the writings of Dr. Russ alike to the official and unofficial worker in social reform.

* Published by the Scientific Press, Ltd., Southampton Street, Strand.

